# Microplastics matter: practical implications of the EU REACH restriction for the pharmaceutical industry

**DOI:** 10.3389/fddev.2026.1854462

**Published:** 2026-06-04

**Authors:** Pauline Henrica Maria Janssen, Willem Antoon Rijpkema, Lorina Bisharat, Marlijn Orbons, Maarten Jaspers, Bastiaan Hendricus Jozef Dickhoff

**Affiliations:** DFE Pharma GmbH & Co. KG, Innovation and Technical Services, Goch, Germany

**Keywords:** biodegradability, medicinal product manufacturing, microplastics, polymeric excipients, reach, regulatory science, sustainability, synthetic polymer microparticles

## Abstract

Synthetic polymer microparticles (SPMs), commonly referred to as microplastics, are subject to increasing restrictions due to their environmental persistence and uncertainty about potential human health effects. These substances are regulated under Regulation (EC) No 1907/2006 (REACH). Commission Regulation (EU) 2023/2055 amends REACH by introducing a restriction on SPMs in Annex XVII, including phased information and reporting obligations for in-scope uses. Although medicinal products are derogated from the market ban, these obligations have practical implications for pharmaceutical supply chains. Medicinal products containing SPMs are subject to specific reporting obligations under the REACH regulation. These obligations apply when the excipients meet the SPM definition and cannot be excluded based on criteria such as being naturally occurring, or due to their solubility or demonstrated degradability. This paper presents an operational decision-making framework that interprets the EU REACH restriction on SPM for pharmaceutical excipients and drug-delivery systems. The framework integrates regulatory definitions with testing strategies, supplier qualification processes, and downstream compliance workflows. An industry case example illustrates a combined approach using portfolio screening and biodegradation testing. This can generate evidence to support compliance decisions while preserving formulation performance.

## 1 Introduction: why microplastics matter

Microplastics are small solid plastic particles, typically smaller than 5 mm, originating either from intentionally manufactured microscopic materials or from the breakdown of larger plastic items (European Commission, 2025a; Song et al., 2024). Microplastics are durable and do not biodegrade under natural conditions. As a result, they accumulate in ecosystems, causing long-term pollution.

Although scientific understanding is still developing, early studies suggest links to inflammation, oxidative stress, and cellular damage (Li et al., 2026; Choudhary et al., 2025). Because of their size, microplastics can be ingested and may cause physical blockages, reduced feeding, inflammation, impaired reproduction, and increased mortality (Ali et al., 2024; Lamoree et al., 2025). The extent of human health risk is still unknown, keeping microplastics a priority topic for global research and regulation (Nayak et al., 2026; Tang et al., 2024).

Governments and international organizations are acting to reduce microplastic emissions. The European Union (EU), for example, aims for a 30% reduction of microplastics released into the environment by 2030 (European Commission, 2023a). To support this target, the EU has introduced a restriction under Regulation (EC) No 1907/2006 (REACH), as amended by Commission Regulation (EU) 2023/2055 (European Commission, 2023b). This restriction prohibits placing on the market of substances and mixtures containing intentionally added synthetic polymer microparticles (SPMs) that are inevitably released during use. Certain sectors, like medicinal products and excipients used at industrial sites, are excluded from the ban because of the criticality of those products (U.S. Congress, 2015). Sectors with such an exception, however, remain subject to information and reporting obligations.

Polymers are widely employed in the pharmaceutical industry as excipients to adjust the performance of medicinal products. Superdisintegrants, film coatings, binders, controlled-release agents, and viscosity modifiers are typically polymeric excipients (Arun et al., 2024). Polymeric excipients may trigger SPM-related obligations where they meet the SPM definition and cannot be excluded on the basis of being naturally occurring and not chemically modified, or on the basis of solubility or demonstrated degradability.

The reporting requirements introduced by the EU REACH SPM restriction pose compliance challenges for medicinal product manufacturers. Estimating annual emissions of SPMs can be complex, as such estimates rely on lifecycle-based assumptions. This typically requires extensive analysis, especially in the absence of standardized methodologies or historical reference data (Maga et al., 2022). Furthermore, reporting must be prepared in IUCLID^1^ format, and the dossier should be submitted to the European Chemicals Agency (ECHA) via REACH-IT^2^, which lies outside the traditional pharmaceutical regulatory framework (European Chemicals Agency, 2025). To facilitate enforcement, the reported information is made available by ECHA to competent member state authorities. National authorities oversee and enforce regulations on chemicals and environmental protection. Examples include ANSES^3^ in France or Umweltbundesamt (UBA)^4^ in Germany (European Commission, 2023b). Consequently, information requests are handled through environmental departments rather than regulatory affairs or quality assurance functions, requiring adapted internal processes and stronger data governance.

This manuscript introduces a novel, practice-oriented framework that advances the pharmaceutical sector’s approach to REACH compliance. While current ECHA and IPEC guides largely focus on regulatory definitions and high-level reporting considerations, this work distills the essential REACH SPM definitions and translates exclusion criteria into a structured, actionable decision workflow tailored to excipient grade assessments for pharmaceutical applications. The framework links regulatory requirements to practical, tiered test strategies and clarifies responsibilities for downstream communication between excipient suppliers and medicinal product manufacturers.

These methodological contributions are complemented by a fully worked industry case example, demonstrating a strategy that combines portfolio screening with targeted biodegradation testing. This case illustrates how evidence can be generated while preserving formulation integrity and supporting efficient compliance decision-making. Collectively, these contributions establish a new benchmark for operationalizing SPM regulations within the pharmaceutical sector, moving beyond the limitations of existing guidelines to deliver a comprehensive and industry-adapted compliance strategy.

## 2 Scope

This review is structured as a narrative review and adopts a practice-oriented approach. It distills the REACH legal text and associated explanatory guide and reporting guidelines cited herein, together with selected peer-reviewed literature on microplastics and biodegradation testing of polymeric excipients. It clarifies the decision points that determine whether a formulation component may trigger SPM-related obligations. It also outlines methodological considerations for generating defensible evidence in a regulated pharmaceutical context. A detailed categorization of all polymer classes used in drug delivery is out of scope.

The primary sources for this review were regulatory texts and associated guidance documents. Additionally, a literature search was conducted in a structured, but non-systematic manner. Google Scholar was used as the primary starting point to identify peer-reviewed articles regarding polymer biodegradability, SPM regulations, and pharmaceutical excipients. Search terms included combinations of “synthetic polymer microparticles”, “microplastics”, “biodegradability”, “REACH”, and “pharmaceutical excipients”. Titles and abstracts were screened for relevance to oral solid dosage applications and regulatory decision-making. Publications were selected based on their relevance to excipient classification, test methodology, or regulatory interpretation rather than publication volume alone. This approach prioritized practical applicability and regulatory relevance over exhaustive coverage of the broader biodegradation literature.

Although this is a narrative review, the case study example followed a structured, reproducible screening workflow to ensure regulatory robustness and practical transferability. The case study started with listing the entire portfolio of DFE Pharma. This list was extended with commonly used excipients, selected based on their reported prevalence in marketed oral solid dosage forms (U.S. Food and Drug Administration, 2026; Jivraj et al., 2000). Material chemistry and production process were reviewed first. Thereafter, physicochemical properties particle size and solubility were evaluated. Polymers of the DFE Pharma portfolio that were still in scope of step 3 were reviewed on biodegradability.

## 3 The EU REACH SPM restriction: relevance for pharmaceutical products

In September 2023, the EU adopted Commission Regulation (EU) 2023/2055 (European Commission, 2023b), amending Regulation (EC) No 1907/2006 (REACH) by introducing a restriction on SPMs that are intentionally added to products. This amendment applies across multiple sectors through a combination of placing-on-the-market bans, derogations, and transitional measures, with obligations phased in following its entry into force in 2023.

### 3.1 Definition and boundary conditions

Polymers are long-chain molecules made up of repeating units that are distributed over a range of molecular weights (Callister and Rethwisch, 2018). Under REACH, polymers are considered SPMs if they are solid and fulfill both of the following conditions (European Commission, 2025a):

- are contained in particles and constitute at least 1% w/w of those particles; or build a continuous surface coating on particles;
- at least 1% w/w of the particles referred to in point (a) fulfill either of the following conditions:
  - all dimensions of the particles are equal to or less than 5 mm;
  - the length of the particles is equal to or less than 15 mm and their length to diameter ratio is greater than three.

Many small polymer beads, powders, or fragments used in pharmaceutical products are classified as SPMs under this definition.

### 3.2 Exclusions and derogations: why evidence matters

Under the REACH SPM restriction, the following polymers are excluded from the designation of SPM (European Commission, 2025a):

- polymers that are the result of a polymerization process that has taken place in nature, independently of the process through which they have been extracted, which are not chemically modified substances;
- polymers that are degradable as proven in accordance with European Commission (2025a);
- polymers that have a solubility greater than 2 g/L in water as proved in accordance with European Commission (2025a);
- polymers that do not contain carbon atoms in their chemical structure.

These exclusions are relevant for pharmaceutical excipients, as many materials are either non-polymeric or qualify as water-soluble polymers, biodegradable, or naturally occurring polymers that have not been chemically modified.

**FIGURE 1:**
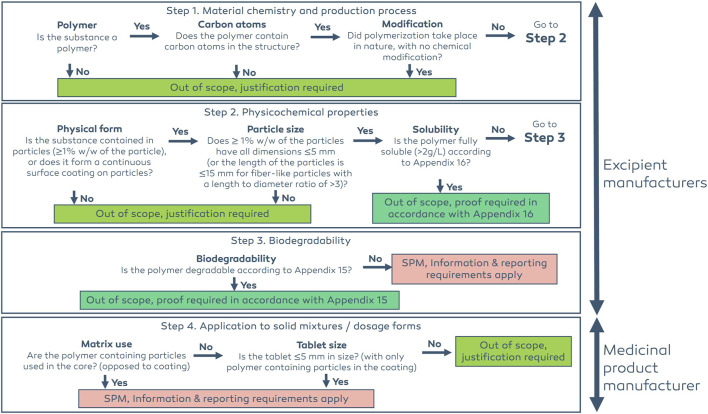
Four-step decision workflow translating the REACH microplastics restriction into an excipient qualification pathway. Steps 1 focus on intrinsic material properties, related to the chemistry, physicochemical properties and the biodegradability. Step 4 describes the application of SPM criteria to dosage forms.

We propose a four-step implementation workflow to review the SPM restriction applicability, as visualized in Figure 1. The first step involves screening of the material chemistry and production process of the substance. The key outcome is whether the material needs a justification document or needs to be further evaluated. The second step focuses on the physicochemical properties. Relevant inputs include solubility evidence as per European Commission (2025a) and particle size measurement data. The decision to be made is whether exclusion justification can be defended for the specific grade and supplier. Step three addresses biodegradability assessment in accordance with European Commission (2025a). The decision to be made is if exclusion based upon biodegradability data is justified. Steps one to three focus on the evaluation of the excipient itself.

Tablets are considered particles under the restriction. If a polymer qualifies as SPM in steps 1 to 3, the application in the dosage form should be reviewed as defined in step 4. In this step it is determined if information and reporting requirements apply. Part I, Section 2 of the explanatory guide and IPEC best practices guide illustrate how these SPM criteria are implemented for tablets or capsules containing internal granules and/or coatings. The guides provide additional illustrative examples and contextual explanation (IPEC Europe, 2025; U.S. Congress, 2015). This workflow, shown in Figure 1, separates the evaluation of intrinsic material properties in steps 1-3 from application to dosage forms considerations in step 4, in order to support early-stage excipient qualification (IPEC Europe, 2025).

Specific thresholds are established for most parameters to determine exclusion eligibility. European Commission (2025a) outline detailed testing conditions, validity conditions and the respective thresholds for biodegradability and solubility assessments (Organisation for Economic Co-operation and Development, 1992a). Explicit consideration of result uncertainty is, however, essential, particularly when outcomes are near the decision threshold. Uncertainty may arise from limitations in analytical methods, laboratory variability, and the representativeness of the tested sample compared to the marketed grade. When data is close to the defined threshold, decisions should be substantiated with additional supporting evidence, such as replicate measurements or confirmatory tests, which must also be reported. In situations where there is uncertainty about whether a material meets the SPM definition or qualifies for an exclusion, it is advisable to apply a cautious, conservative approach. Medicinal product manufacturers should, for example, rely on evidence provided specifically for the polymer grade or excipient supplier in question. Evidence from other suppliers or grades should only be used if their comparability has been clearly demonstrated.

### 3.3 Regulatory implications for pharmaceutical development and manufacturing

#### 3.3.1 Placing-on-the-market ban

For many product categories, REACH introduces restrictions on substances and mixtures containing SPMs at a concentration threshold of 0.01% w/w. These restrictions may necessitate reformulation, with phase-in timelines that depend on product type (European Commission, 2023b; Coslaw, 2023). Medicinal products are exempt from the placing-on-the-market ban, reflecting the essential role of polymers in dosage forms and the need to safeguard continuity of supply. However, this exemption does not eliminate all regulatory obligations. REACH information and reporting requirements must still be met.

#### 3.3.2 Information (including instructions for safe use and disposal) requirement

From October 2025 onwards, the information obligations apply to certain actors placing SPMs on the EU market. Where applicable, suppliers must ensure that users have access to specified information, including polymer identity, quantity or concentration, and instructions for safe use and disposal to minimize environmental release (European Commission, 2023b; U.S. Congress, 2015). While medicinal products are not subject to additional instructions for use and disposal under REACH, these provisions may still affect upstream actors in the pharmaceutical supply chain. For medicinal product manufacturers, this translates into earlier and more structured information requests to excipient and component suppliers, and into maintaining documentation in a manner that allows information to be linked to specific formulation versions and suppliers.

#### 3.3.3 Reporting requirement

For derogated applications, including medicinal products, reporting to ECHA is expected to start for the calendar year 2026, with the first submission due by 31 May 2027 via IUCLID/REACH-IT (European Chemicals Agency, 2025). Reports must include a description of the relevant use, generic polymer identity information (e.g., polymer type), and an estimate of annual emissions to the environment. Product or use categorization may additionally be supported by HS/CN codes, consistent with ECHA reporting guidance. Where available, supporting information such as pharmacopeial specifications or indicative particle size ranges may be relevant. For oral medicinal products, potential environmental release may primarily arise from disposal pathways, such as unused or expired medicines (Organisation for Economic Co-operation and Development, 2022). Nonetheless, consistent reporting requires the use of defensible assumptions and transparent data provenance to support reproducibility and year-on-year tracking.

### 3.4 Global and future implications

Although the REACH restriction on SPMs is EU-based, it can affect global pharmaceutical supply chains. The obligations apply to products placed on the EU market, including those supplied by non-EU manufacturers (European Commission, 2025b; U.S. Congress, 2015). As a result, polymer declarations and exclusion justifications may increasingly become standard documentation requests across regions, particularly for excipients and drug delivery components that are broadly distributed. While the requirements are regulatory in nature, they also have direct implications for formulation design and drug delivery performance, as discussed in Section 6.

Beyond the EU, approaches to regulating microplastics are developing but remain diverse. In the United States, regulation has largely focused on specific products and sectors, most notably through the federal ban on plastic microbeads in rinse-off cosmetics (U.S. Congress, 2015). Broader action on microplastics is pursued mainly through a combination of state-level measures and federal research and monitoring initiatives. In parallel, U.S. FDA and U.S. Environmental Protection Agency are actively monitoring microplastics in food, water, and the environment through research and surveillance programs, without introducing binding restrictions. The United Kingdom has taken a similar targeted approach (UK Government, 2017), while operating its own chemicals framework under UK REACH that does not currently include a horizontal restriction on intentionally added microplastics equivalent to EU REACH Annex XVII Entry 78. Canada also has a product-focused approach with prohibitions in for example toiletries (Government of Canada, 2017), while prescription drugs are excluded for now. China also operates with product-specific restrictions to microplastics, mainly focused on cosmetics. Those restrictions are supplemented by analytical standards and broader environmental policy instruments addressing plastic pollution and emerging pollutants (State Administration for Market Regulation SAMR and Standardization Administration of China SAC, 2021). In India, microplastics are increasingly addressed through broader plastic-waste and environmental policy developments, with ongoing discussion on definitions and controls for intentionally added microplastics alongside wider single-use plastics measures (Government of India and Ministry of Health and Family Welfa re, 2020).

From a harmonization perspective, the main future challenges are likely to relate less to the general policy objective and more to technical boundary conditions. Such challenges may arise from potential differences in legal definitions and thresholds, varying exemption and derogation structures, and non-aligned expectations for evidence generation should similar regulatory approaches be introduced or expanded across regions. If such regimes emerge, divergent reporting formats and data systems could further increase the burden for global supply chains, as the same polymeric excipient may require jurisdiction-specific classifications, datasets, and dossier outputs. Accordingly, while our decision workflow is grounded in EU REACH, its stepwise structure is intended to be transferable by substituting jurisdiction-specific definitions, exclusion criteria, and documentation endpoints.

The regulatory landscape for microplastics continues to evolve. Exemptions and derogations may be revisited as policymakers reassess risks and emission inventories. In parallel, other jurisdictions may introduce similar requirements (United Nations Environment Programme and Intergovernmental Negotiating Committee on plastic pollution, 2022), further increasing the complexity of global compliance. As a result, excipient suppliers and market authorization holders need to remain proactive and flexible, recognizing that the current regulatory framework may change as international policies develop.

## 4 Practical expectations for excipient suppliers and medicinal product manufacturers

Pharmaceutical excipient suppliers have a significant role in supporting medicinal product manufacturers in achieving regulatory compliance. Suppliers are expected to screen their portfolio, provide generic polymer identity information, and generate evidence to support exclusion from the SPM designation where applicable. Transparent sharing of data packages is essential to enable continued use of polymeric excipients in medicinal products while complying with environmental regulations. Suppliers that can demonstrate that their excipients do not fall within the scope of the REACH SPM restriction can effectively remove a limitation for formulators and will be preferred in an era of rising sustainability expectations.

### 4.1 Exclusion justification and data traceability

Polymeric excipients may be exempt from regulatory oversight if they fulfill any of the specific exclusion criteria. Where an exclusion is claimed, suppliers should provide clear justification supported by test reports or other verifiable evidence aligned with the REACH appendices (European Commission, 2025b; U.S. Congress, 2015). Because polymeric excipients can vary by source materials and processing conditions (Dave et al., 2015; Narang, 2015), exclusion evidence should be linked to the specific grade and manufacturing site where relevant and communicated in a format that downstream users can archive and reference when relevant authorities request it.

### 4.2 Managing in-scope materials: information and reporting readiness

For materials that are not proven to be out of scope, raw material suppliers need to act. Safety data sheets and technical documentation must be revised to incorporate pertinent instructions regarding handling and disposal. A clear indication of the material’s classification as a SPM must also be included.

To support reporting, suppliers must deliver precise details about SPM identity, including the chemical name, polymer classification, particle size distribution, and relevant environmental fate data. Supplying accurate, timely information assists pharmaceutical companies in estimating emissions, justifying regulatory submissions, and responding to ECHA reporting requirements from May 2027 onwards (European Commission, 2023b).

### 4.3 Best practices: supplier variability and comparability

When a formulation component is sourced from multiple suppliers, exclusion status should be evaluated per supplier and per grade. Even where the same pharmacopeial name and CAS number are used, polymer attributes (e.g., degree of substitution, cross-linking density, molecular weight distribution, and particle morphology) may differ, which influences solubility and degradability (Bading et al., 2024a; Shah and Augsburger, 2002; Zhao and Augsburger, 2006). For example, sodium starch glycolate from different companies can vary in degree of carboxymethyl substitution and cross-link density (Shah and Augsburger, 2002). Likewise, croscarmellose sodium made from different botanic sources can have different molecular weights (Zhao and Augsburger, 2006). Consequently, documentation from one supplier cannot automatically be assumed applicable to another without an explicit comparability rationale.

This need for supplier-specific evidence parallels the nitrosamine concern, where materials meeting the same specifications can differ in nitrite levels (Boetzel et al., 2023). For REACH SPM compliance, early alignment between suppliers and manufacturers on data requirements and change notification expectations may reduce the likelihood of late-stage reformulation or reporting uncertainty.

## 5 Industry case example: portfolio screening and biodegradability evidence generation for polymeric excipients

To illustrate a pragmatic approach for addressing EU REACH restriction on SPM for pharmaceutical excipients, this section summarizes an industry case example focused on polymeric excipients. The approach combines (i) systematic portfolio screening of an excipient supplier against the SPM definition and exclusions, (ii) targeted testing aligned with REACH European Commission (2025a) where evidence is needed, and (iii) structured documentation to support downstream users’ compliance workflows.

### 5.1 Initial screening: identifying materials that warrant further assessment

In the case study, the portfolio-level assessment of an excipient supplier began by categorizing materials as polymers or non-polymers, then determining whether those polymers contained carbon atoms and if they were chemically altered as described in step 1 of Figure 1. For our case example, initial evaluation revealed that most excipient groups could be exempted from further consideration.

Disaccharides such as sucrose, trehalose, and lactose, are excluded as they are considered small organic molecules rather than polymers. Other excipients that are not polymers include mannitol, sorbitol, dicalcium phosphate, magnesium stearate, silicon dioxide, and sodium stearyl fumarate.

Natural polymers that have not been chemically modified are also excluded, which are for example microcrystalline cellulose, native potato starch, and pregelatinized starch. Chemical modification in this sense refers to the introduction of new chemical substituents. This does not occur in the case of pregelatinized starch, as this undergoes physical processing using heat and/or moisture to disrupt its granular structure, which does not lead to chemical derivatization or functional group substitution (Rowe et al., 2012). Similarly, microcrystalline cellulose is not produced by the introduction of new chemical substituents, as this is produced through controlled hydrolysis of cellulose (natural polymer) (Hindi, 2017). Even though microcrystalline cellulose is commonly treated as a naturally occurring polymer, the hydrolysis might raise the question whether such processing should still be regarded as “not chemically modified”. Should MCC not be accepted under the “not chemically modified” criterion, exclusion would need to be demonstrated through subsequent evaluation steps. Step 2 would not provide any exclusion for MCC, as MCC particles typically fall within the SPM size range and are insoluble. Biodegradability testing as defined in step 3 would therefore be appropriate. MCC is widely recognized as biodegradable and is commonly used as a reference material in biodegradation studies, providing a strong scientific basis to substantiate its degradability when required (European Commission, 2023b; Hubbe et al., 2025; Saha et al., 2019).

Chemically modified polymers include for example sodium starch glycolate, croscarmellose sodium, hydroxypropyl methylcellulose, hydroxypropyl cellulose, ethyl cellulose, polyethylene glycol, polyvinyl pyrrolidone, copovidone and crospovidone. Without explicit proof of physicochemical exclusion or biodegradability, those products may fall within the definition of SPMs, making them subject to restriction. Bading et al. reviewed different polymeric compounds in an experimental set-up on biodegradability (Bading et al., 2024b). Polyvinyl alcohol, polyethylene glycol, and chitosan were identified as readily biodegradable. In contrast, polyvinyl pyrrolidone, copovidone, and crospovidone were categorized as non-biodegradable, explained by the lack of oxidizable/hydrolysable groups within their carbon backbone structure.

Within our case study, two cross-linked, carboxymethylated superdisintegrants emerged as candidates for step 2 evaluation. Primojel® is a sodium starch glycolate type A with a particle size of min. 100% w/w < 125 µm. It consists of glucose-based polymers, originating from potato starch. Those polymers have been chemically modified (carboxymethylated and cross-linked), making them insoluble (Manzoor, 2021; Janssen et al., 2025). Primellose® is croscarmellose sodium with a particle size of min. 98% < 125 µm. It consists of glucose-based polymers, originating from cotton-based cellulose. Those polymers have also been carboxymethylated and cross-linked, making them insoluble (Zhao and Augsburger, 2006; Berardi et al., 2022). Both superdisintegrants therefore were evaluated on biodegradability, as defined in step 3.

### 5.2 Generating degradability evidence: selecting fit-for-purpose tests

Where degradability data are required to support exclusion, REACH European Commission (2025a) provides a tiered framework of accepted test methods and pass criteria (U.S. Congress, 2015; Bading et al., 2024a). Across the European Commission (2025a) test groups, a polymer is considered degradable if it passes any of several tests in one of the five testing groups. Methods range from screening assessments of mineralization (CO_2_ evolution) to higher-tier inherent biodegradability tests and environmental simulation studies (Organisation for Economic Co-operation and Development, 1992a). It is important to note that biodegradability studies conducted in accordance with European Commission (2025a) and under good laboratory practice (GLP) principles often demand significant resources and may take several weeks or even months to finish. When more advanced simulations are needed, testing could last up to 24 months. These realities make rapid screening unfeasible, so prioritization is necessary to prevent delays later in excipient qualifications and ensure timely reporting downstream.

While standardized laboratory tests provide a controlled framework for assessing biodegradability, they are not without limitations. Variability in test conditions, including microbial inoculum, temperature, and nutrient composition, can lead to inconsistent results across laboratories, complicating direct comparison between studies (Howard and Banerjee, 1984). This challenge is mitigated by specified validity criteria in the OECD guidelines (Organisation for Economic Co-operation and Development, 1992b). Additionally, the environmental relevance of such tests is often questioned, as laboratory protocols may not accurately reflect the complexity of real-world ecosystems where pharmaceutical excipients are ultimately released (Dalmijn et al., 2021; Tian et al., 2023). Uncertainties arise when extrapolating laboratory mineralization rates to field scenarios, since factors like soil composition, water flow, and microbial diversity can significantly alter degradation behavior (Martin-Aparicio et al., 2023). Therefore, while lab-based evidence is essential for regulatory compliance, it should be interpreted with caution and supplemented by environmental fate studies when possible.

#### 5.2.1 OECD 301B CO_2_ evolution testing

In the case example, Primojel® and Primellose® required biodegradability evaluation. Both products were evaluated using a CO_2_ Evolution Test (OECD 301B) conducted by an accredited external laboratory under GLP conditions (Organisation for Economic Co-operation and Development, 1992b). Reported mineralization was 63.8% of theoretical CO_2_ content (ThCO_2_) for Primojel® and 75.5% of ThCO_2_ for Primellose® at 60 days (mean of three replicates).

The results met the European Commission (2025a) criteria for group 2 screening, achieving ≥60% mineralization in 60 days for both products. This justifies an exclusion claim under Regulation (EU) 2023/2055 for both products.

### 5.3 Documentation deliverables for downstream users

For pharmaceutical users, the practical value of exclusion evidence depends on how easily it can be incorporated into operational processes. Data packages typically need to be version-controlled, supplier-specific, and aligned with internal material master data so that formulation changes, supplier switches, and site transfers can be assessed for potential impact on SPM-related obligations.

In the case example, downstream-support documentation included (i) a portfolio-level overview summarizing SPM screening outcomes (Table 1) and (ii) detailed biodegradability test reports for the assessed superdisintegrants. Such documentation can help manufacturers substantiate internal decisions, respond to customer or authority questions, and maintain consistency across reporting cycles as implementation guidance evolves.

**TABLE 1:** Results from the case study on DFE Pharma’s portfolio evaluation of product status as SPM. Green V's indicate which criteria justified that the product is not considered to be an SPM.

DFE pharma product	Potential SPM?	No polymer? (V/X)	No carbon atoms in structure? (V/X)	Not chemically modified? (V/X)	Outside size range (≤5 mm) (V/X)	Soluble (>2 g/L)? (V/X)	Biodegradable? (V/X)
BioHale® Sucrose	No	V					
BioHale® Trehalose	No	V					
Pharmatose®, Lactochem®, SuperTab®, Lactopress® (Lactose)	No	V					
Pharmacel® (Microcrystalline Cellulose)	No	X	X	V?	X	X	V
Nutracel® (Microcrystalline Cellulose)	No	X	X	V			
Solani amylum (Potato Starch)	No	X	X	V			
Prejel PA5 PH (Pregelatinized Starch)	No	X	X	V			
Primojel® (Sodium Starch Glycolate)	No	X	X	X	X	X	V
Primellose® (Croscarmellose Sodium)	No	X	X	X	X	X	V

## 6 Conclusions and future directions for drug delivery materials

The REACH SPM restriction introduces a new compliance dimension for pharmaceutical development, particularly for polymeric excipients and drug delivery components that could meet the SPM definition. Although medicinal products are exempt from the placing-on-the-market ban, supply chains remain subject to phased information and reporting obligations.

SPM regulations increase formulation complexity because it may call for excipient substitution. This may include reformulation towards alternative chemistries or towards grades with different functional related characteristics. Potential SPM materials are chemically modified polymer excipients, which are typically disintegrants and controlled release agents, where functionality depends on the exact polymeric structure. Changes in the grade can affect disintegration, drug release, and process robustness. Therefore, SPM-related decision-making should be integrated early into formulation design and supplier qualification, alongside traditional quality-by-design activities, to avoid late-stage reformulation and to enable timely, reproducible reporting. The case example illustrates how portfolio screening plus targeted biodegradability testing can be used to generate defensible documentation without framing compliance solely as a reformulation problem.

Looking ahead, several areas for future research have been identified that would strengthen both scientific confidence and regulatory robustness for REACH SPM implementation in pharmaceutical applications. First, there is a need to develop practical, tiered testing strategies that enable rapid screening of excipient grades. In parallel, defensible methodologies are required to justify when data can be bridged across polymer grades, manufacturing sites, and suppliers. This will require clearer identification of the relevant material attributes that govern solubility and degradability behavior.

From a regulatory perspective, a key practical bottleneck remains the prediction of emissions for medicinal products, particularly where releases are driven by downstream disposal pathways. Greater harmonization of reporting assumptions, including explicit treatment of uncertainties, would help reduce divergent interpretations across organizations. Finally, future developments in microplastics regulation would benefit from closer alignment with existing EU REACH frameworks. Authorities introducing new or additional microplastics-specific requirements should leverage and harmonize with the extensive work already undertaken at EU level, including centralized reporting and control mechanisms.

Coordinated efforts between excipient suppliers, medicinal product manufacturers, and regulators will be essential to sustain polymer innovation in drug delivery, while meeting emerging environmental expectations.

## Footnotes

1 International Uniform Chemical Information Database

2 European Chemicals Agency’s secure regulatory portal

3 French National Agency for Food, Environmental and Occupational Health Safety

4 German Environment Agency
